# UNDERSTANDING THE LONG-TERM EFFECTS OF PUBLIC OPEN SPACE ON OLDER ADULTS’ FUNCTIONAL ABILITY AND MENTAL HEALTH

**DOI:** 10.1093/geroni/igad104.0906

**Published:** 2023-12-21

**Authors:** Terry Lum, Yuqi Liu, Yingqqi Guo, Shiyu Lu, On Fung Chan, Cheryl Chui, Hung Chak Ho, Yimeng Song

**Affiliations:** The University of Hong Kong, Hong Kong, Hong Kong; South China University of Technology, Guangzhou, Guangdong, China (People’s Republic); Hong Kong Baptist University, Hong Kong, Hong Kong; City University of Hong Kong, Kowloon, Hong Kong; The University of Hong Kong, Hong Kong, Hong Kong; The University of Hong Kong, Hong Kong, Hong Kong; The University of Hong Kong, Hong Kong, Hong Kong; The Hong Kong Polytechnic University, Hong Kong, Hong Kong

## Abstract

Little is known about how public open space (POS) environment quality and vitality influence older adults’ functional ability and mental health over time. POS vitality refers to the capacity of POS to accommodate a variety of users and activities. We undertook a four-year longitudinal survey of 2081 older adults in Hong Kong to investigate longitudinal relationships between POS environment quality, POS vitality, functional ability and mental health. We applied environment quality evaluation and space use behavior observation to collect data on the environment quality and vitality of POSs within the 200-m buffer area of participants’ residences. POS environment quality attributes included the number of leisure facility types, accessibility, shade, and bench quality. POS vitality attributes comprised the diversity of users and activities. We used the Chinese Lawton Instrumental Activities of Daily Living Scale to measure older adults’ functional ability and the Geriatric Depression Scale (15-item) to evaluate mental health. We applied latent growth curve models to analyze the longitudinal associations. Accessibility to POS and social interactions among users in POS were related to better functional ability and mental health among older adults at baseline. The number of leisure facility types, and social interactions among users in POS led to a slower decline in functional ability over time. However, there were no significant associations between POS and mental health over time. These findings have theoretical implications for the healthy aging research framework and practical insights for planning policies using POS as an intervention tool to facilitate older adults’ healthy aging.

